# Control of Respiratory Motion by Hypnosis Intervention during Radiotherapy of Lung Cancer I

**DOI:** 10.1155/2013/574934

**Published:** 2013-09-04

**Authors:** Rongmao Li, Jie Deng, Yaoqin Xie

**Affiliations:** ^1^Institute of Biomedical and Health Engineering, Shenzhen Institutes of Advanced Technology, Chinese Academy of Sciences, 1068 Xueyuan Anenue, Shenzhen University Town, Shenzhen 518055, China; ^2^Zhuhai Psychological Counseling Co., Ltd, 201-40A Haisong Building, 9th Tairan Road, Shenzhen 518000, China; ^3^Lab for Wearable Devices, Key Lab for Health Informatics of Chinese Academy of Sciences, 1068 Xueyuan Anenue, Shenzhen University Town, Shenzhen 518055, China

## Abstract

The uncertain position of lung tumor during radiotherapy compromises the treatment effect. To effectively control respiratory motion during radiotherapy of lung cancer without any side effects, a novel control scheme, hypnosis, has been introduced in lung cancer treatment. In order to verify the suggested method, six volunteers were selected with a wide range of distribution of age, weight, and chest circumference. A set of experiments have been conducted for each volunteer, under the guidance of the professional hypnotist. All the experiments were repeated in the same environmental condition. The amplitude of respiration has been recorded under the normal state and hypnosis, respectively. Experimental results show that the respiration motion of volunteers in hypnosis has smaller and more stable amplitudes than in normal state. That implies that the hypnosis intervention can be an alternative way for respiratory control, which can effectively reduce the respiratory amplitude and increase the stability of respiratory cycle. The proposed method will find useful application in image-guided radiotherapy.

## 1. Introduction

A prerequisite of treatment planning in thoracic radiotherapy is the accurate modeling of respiratory motion of thoracic structures [1]. Much effort has been devoted to address this issue. One of the traditional methods is that the patients are trained before treatment to make respiration as stable as possible, and the depicted planning target volume (PTV) covers the whole area of tumor motion. Another method is the gating technology, which synchronizes the treatment with the breathing cycle. The radiation at the tumor is applied only during a specific time in the cycle, typically during the deepest expiration of the patient, and the radiation is ceased once the tumor moves out of the targeted area. One of the powerful tools for gating is the respiratory position management (RPM) system [2, 3] developed by Varian, which can detect and track respiratory motion without the need to train the patient. Another respiratory trace generating (RTG) tool was also developed for tracking respiratory motion [4]. The disadvantage of gating technology is the prolonged treatment time. The third method is developed to have the patient breathe regular by a visual system which shows a standard breathing curve and the patient's real-time breathing curve in front of the patient during treatment. The intervention to the patient is inevitable in the course of the treatment, which may result in patient's discomfort. The forth method is real-time tumor tracking. In current image guided radiation therapy (IGRT), it becomes increasingly popular by using implanted metallic or radio frequency fiducials [5–9]. In this approach, stereoscopic X-ray images are taken simultaneously or sequentially during the course of dose delivery. High contrast fiducials are detected on the projection images, and a triangulation algorithm is then employed to extract the positions of the fiducials in real-time. While the fiducial marker provides a reliable way for real time tracking, implantation of fiducial markers is an invasive procedure and may result in a number of possible complications, such as pneumothorax and hemorrhage [10]. 

In this paper, hypnosis is introduced in radiotherapy for respiratory control without any side effects. As we know, hypnosis may keep the patient in inner peace during treatment. Consequently, the respiration amplitude is reduced and becomes stable, which is an ideal state for radiotherapy. We hypothesized that hypnosis can be a helpful tool for respiratory control in various treatment procedures, such as gating and real-time tracking.

## 2. Materials and Methods

### 2.1. Hypnosis

Hypnosis is a state in which a person seems to be asleep but still can see, hear, or respond to speech directed to him [11, 12]. Hypnotherapy is hypnosis used for therapy. Under normal state, people have regular, low-amplitude, high-frequency *α* and uniform-frequency *β* brain waves, while under light hypnosis state, the amplitude of *α*, brain waves becomes higher, and the frequency becomes uniform. Physicians and psychiatrists may use hypnosis to treat depression, anxiety, eating disorders, sleep disorders, compulsive gaming, and posttraumatic stress. Hypnotherapy has been successfully applyied in many cases, such as fears, phobias, habit control, pain management, and psychological therapy. It is widely used in pre- and posttreatment but rarely used during treatment. In this paper, hypnosis is used to make a patient's inner peace and respiration stable during treatment.

To effectively control respiration during radiotherapy of lung cancer, we need to know whether hypnosis works well on stabilizing the respiratory motion and then develop a novel clinical scheme to apply hypnosis in radiotherapy. Several experiments have been conducted to test the influence of hypnosis on respiration. In these experiments, a professional hypnotist guides volunteers into hypnosis state.

Hypnosis technique takes part in an important role in the method. However, the technique is not suitable for all clinical cases. Based on the standard score evaluation, 10% in normal people is highly susceptible population, and 10% is marginally susceptible population. Age, imagination, self-suggestion, and other properties of the subject influence the susceptibility. So the method cannot be applied for all patients. An important work before hypnosis is to determine which patient is suitable for hypnosis therapy. Before hypnosis, several tests should be done to check the susceptibility of the patient and to select the suitable patients. 

### 2.2. Sensors and EEG System

**Figure 1:**
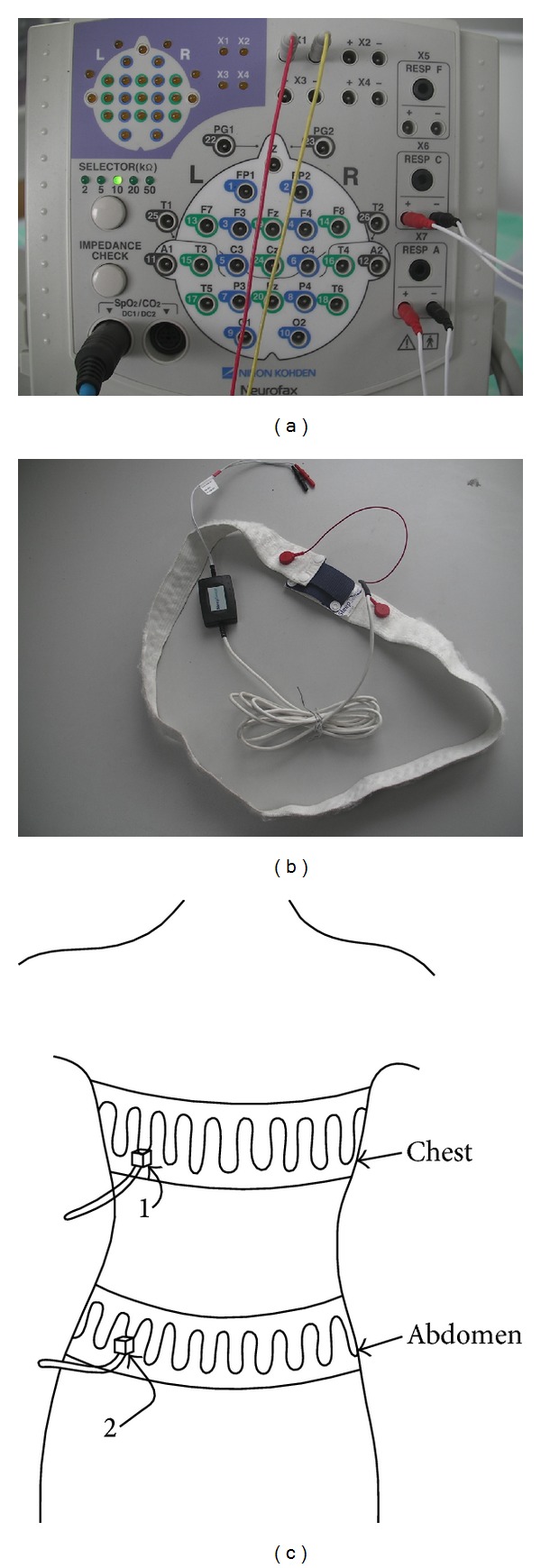
Sensors and EEG system used in the experiment, (a) the EEG system used to record the electrical signal, (b) the sleep sensor used to detect the respiration motion, (c) the schematic diagram of the method to detect the respiration motion.

The electroencephalogram (EEG) system developed by Nihon Kohden Corporation is user-friendly and allows recording the electrical activity of the brain over a short period of time, as shown in Figure 1(a). It consists of a notebook PC, isolation power supply, advanced electrode junction box, and other standard accessories. There is also a full range of optional accessories. Polysmith software is the world's most all-inclusive PSG acquisition and analysis program. Patient data acquisition and analysis are integrated into a single software. As a part of EEG system, it is used in various environments and provides a comprehensive approach to analyze obtained experimental data. It is convenient for remote access from the control room [11].

The sleep sensor in Figure 1(b) is a part of the system that detects the respiration motion. It is tied on the chest and the abdomen of the volunteer. The corresponding signals are transported to the EEG system for further analysis. The inductance of the bands is exactly proportional to the transverse section encircled by the band, as shown in Figure 1(c). The inductance changes with the breathing of the patient. It is converted by the electronic circuits of the system into an electrical signal in order to accurately and reliably record the respiration waveforms. The inductive band can be easily connected to any compatible system through a dedicated interface cable. 

The parameters setting of the sleep sensor is constant in the whole experimental procedure. The sensitivity is 10 *μ*V/mm, the CAL voltage is 50 *μ*V, the time constant is 0.3 s, and the high-cut filter is 50 Hz.

### 2.3. Experimental Procedure

Nine volunteers took part in the experiment. Among them, six volunteers were selected by the hypnotist as suitable subjects for hypnosis with a wide range of distribution of age, weight, and chest circumference, as listed in Table 1. Two experiments have been done with these 6 volunteers. One experiment was conducted under normal breathing. The volunteers peacefully lied on a bed without any motion and speaking for 15 min. Another experiment involved breathing under hypnosis. The hypnotist guided the volunteer into hypnosis state and determined whether the volunteer is under hypnosis. For each subject, it took about 10 min to enter into hypnosis and another 15 min to keep the hypnosis state. Then, the volunteer was awaken by the hypnotist. During the hypnosis procedure, the volunteer was extremely suggestible to the hypnotist. These two experiments were repeated two weeks later.

## 3. Experiment and Results

**Figure 2:**
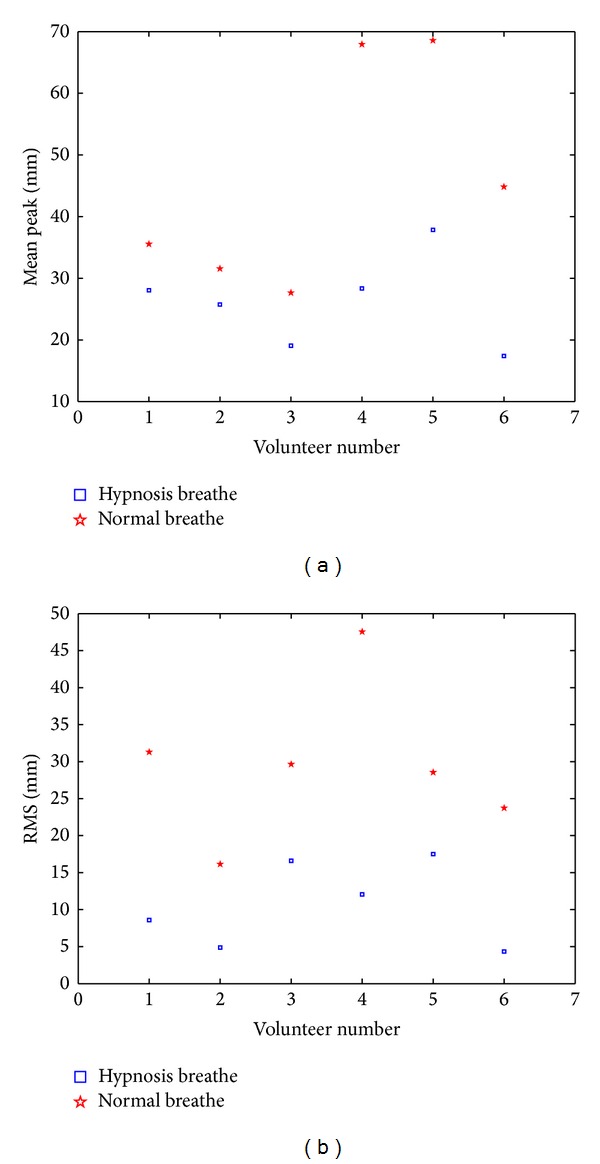
Peak value and maximum root mean square (RMS) of respiratory amplitude for the six volunteers in the normal state (red) and in the hypnosis state (blue), respectively.

Figure 2 shows the mean peak value and the maximum root mean square (RMS) of respiratory amplitude for 6 volunteers in the normal state (red points) and in the hypnosis state (blue points), respectively. The definitions of mean of RMS are

(1) $$\begin{array}{c} \text{Mea}{\text{n}}_{i}\equiv \sum_{j}^{N_{i}}\frac{A_{i}^{j}}{N_{i}},\\ \text{RM}{\text{S}}_{i}=\sqrt{\overset{N_{i}}{\underset{j}{\sum }}{\left(A_{i}^{j}-\text{Mea}{\text{n}}_{i}\right)}^{2}}, \end{array}$$

where *i* is the number of volunteers and *N*_*i*_ is the number of the peaks for volunteer *i*. It is clear from Figure 2 that the peak value in the hypnosis state is much lower than that in the normal state, so as the RMS, indicating more stable respiration in the hypnosis state. In treatment planning, PTV stands for the entire region tumor may cover. The reduction of the mean amplitudes and the RMS is a great help to protect the patient from the dose. The low mean amplitude implies the energy of the radiation more concentrated in the tumor, and the low RMS implies the more stable for treating.

**Figure 3:**
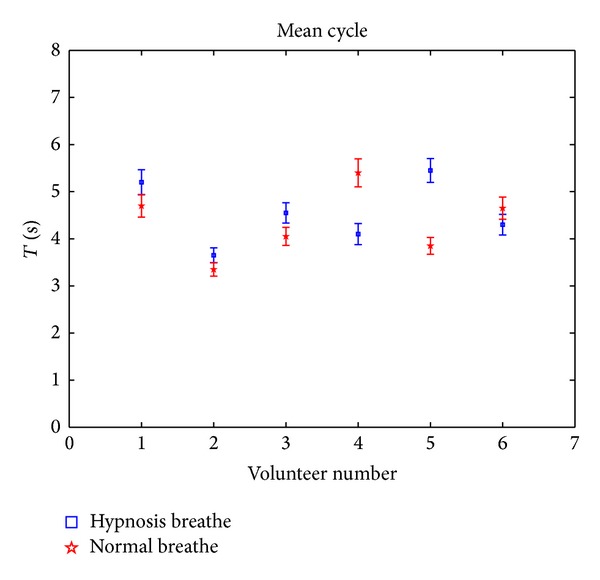
Mean cycle of respiration for different volunteers in the normal state (red) and in the hypnosis state (blue).

Figure 3 shows the mean cycle of respiration of different volunteers under normal state and hypnosis. No obvious difference between these two states is observed. 

**Figure 4:**
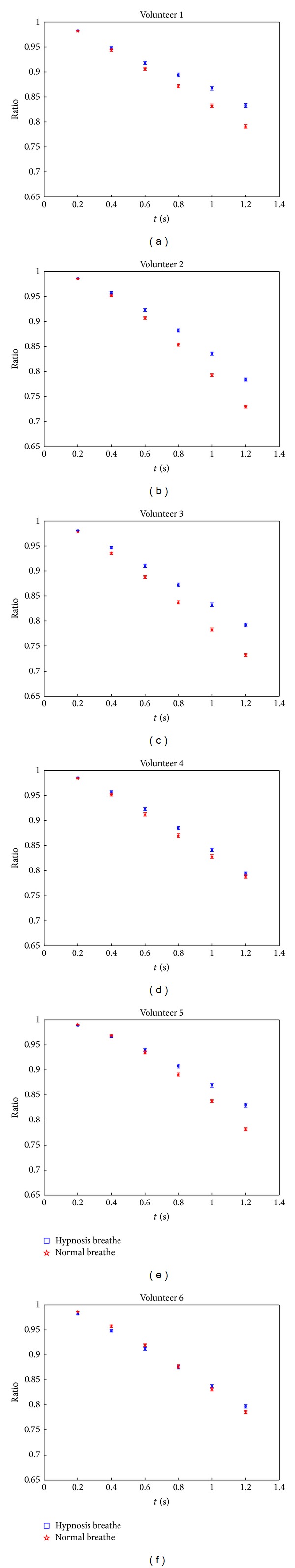
Relative ratio of the wave nearby the peak and the amplitude with time weighted. The red and blue points stand for the normal state and the hypnosis state, respectively.

Figure 4 shows the relative ratio between the amplitude and the time weighted average in a certain range near the peak. The parameter is defined as

(2) $$\begin{array}{c} \text{Ratio}\left(t\right)\equiv \frac{\sum A_{i}t_{i}}{At}, \end{array}$$

where the ratio stands for the displacement around the peak of respiratory waveform. The waveform is divided into many small time intervals, counted by *i*. *t*_*i*_ is the time interval, *A*_*i*_ is the corresponding amplitude of the wave during the time *t*, *A* is the amplitude of the waves, and *t* is the time interval of the wave near the peak. The ratio for each peak is obtained. The final ratio is the mean for all peaks.

In Figure 4, the *x*-axis stands for the time interval nearby the wave peak. It can be seen that the relative ratio in the hypnosis state reduced more slowly than that in the normal state. The slower the relative ratio is reduced, the more stable is the wave nearby the wave peak. 

To evaluate the difference between different cycles, the *γ* index of passing ratio [13–15] is introduced as shown in Figure 6. Like the ratio in (2), *γ* passing ratio is another quantity to evaluate the similarity between different cycles. It is defined as

(3) $$\begin{array}{c} \gamma \left(x_{m}\right)=\min\left\{\Gamma \left(x_{m},x_{c}\right)\right\},\;\forall \left\{x_{c}\right\}, \end{array}$$

where

(4) $$\begin{array}{c} \Gamma \left(x_{m},x_{c}\right)=\sqrt{\frac{x^{2}\left(x_{m},x_{c}\right)}{\Delta d_{M}^{2}}+\frac{\delta^{2}\left(x_{m},x_{c}\right)}{\Delta D_{M}^{2}}},\\ x\left(x_{m},x_{c}\right)=\left|x_{m}-x_{c}\right|,\\ \delta \left(x_{m},x_{c}\right)=D_{m}-D_{c}, \end{array}$$

**Figure 5:**
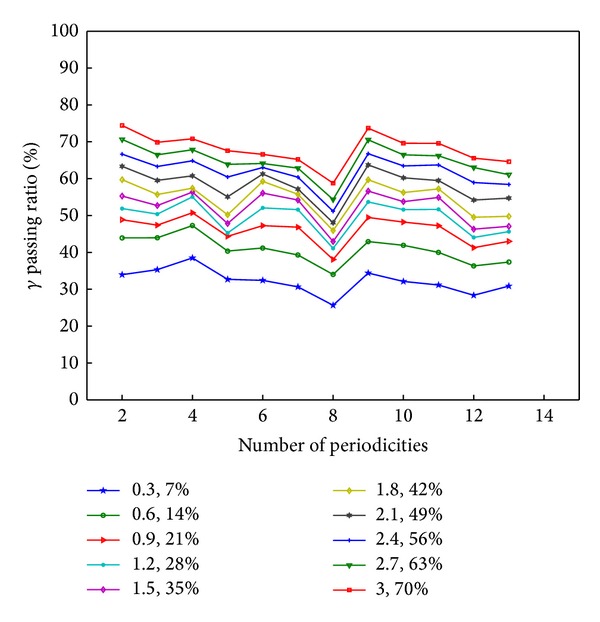
*γ* index of passing ratio between the first cycle and the other 13 adjacent cycles with ten different parameters.

*δ* is the difference between different cycles with *x*_*m*_ and *x*_*c*_. *x*_*m*_ is the time of the template cycle, and *x*_*c*_ is the time of other cycles. In the experiment, the first starting cycle is selected as the template cycle. The *γ* passing ratio is obtained between the template cycle and other cycles. Δ*D*_*M*_ is the parameter to estimate the degree of displacement, Δ*d*_*M*_ is the parameter to estimate the degree of the time to agreement. In our experiments, 10 groups of these two parameters are set to calculate the *γ* passing ratios for different cycles. It can be seen from Figure 5 that the *γ* index is relatively stable in the area of dense curves. Therefore, the parameters of *γ* index are selected as 1.2% and 28%. 

Similar to *γ* passing ratio used in the evaluation in dose distribution, *γ* passing ratio can also be used to estimate the similarity of two respiration cycles. As we know, respiratory motion may result in the change of dose distribution.

**Figure 6:**
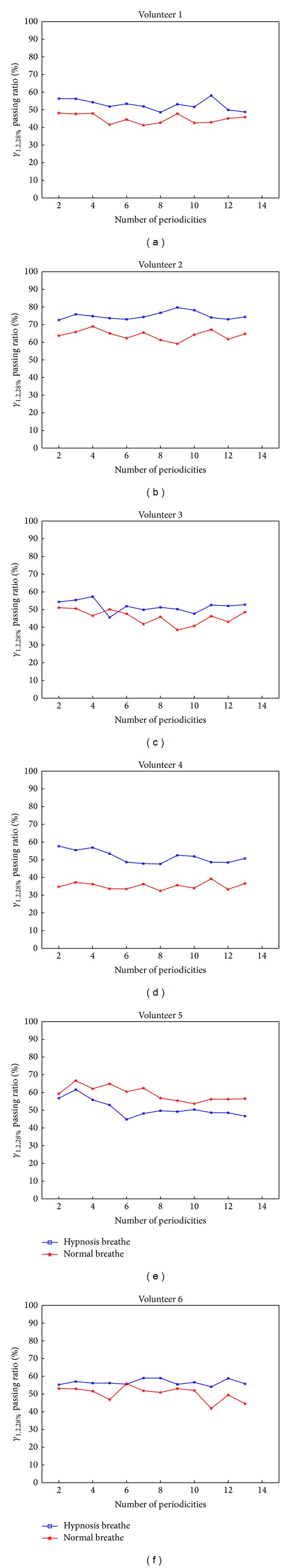
*γ*_1.2,28%_ passing ratio between the first cycle and the next 13 adjacent cycles in the hypnosis state (blue) and the normal state (red).

Figure 6 shows *γ*_1.2,28%_ index curves of passing ratio between the first cycle and the next 12 adjacent cycles in the hypnosis state (blue curves) and the normal state (red curves). Among these 6 volunteers, 5 of them show higher *γ*_1.2,28%  _ passing ratio curves in the hypnosis state than that in the normal state, which means that cycles of respiration in the hypnosis state are much more stable than that in the normal state. The only opposite result as shown in Figure 6 (volunteer 5) indicates that not all the patients are suitable for respiration control using hypnosis.

## 4. Discussions

In this work, a novel method using hypnosis is proposed to control respiratory motion during radiotherapy of lung cancer. Since the hypnosis is comfortable for the patient and makes him peaceful, the method allows treating the patient without side effects during radiotherapy. 

As we know, the traditional training method is widely applied in radiotherapy, which is a benefit to most patients. However, several uncontrollable factors still exist, such as the tension and the surrounding influence during the treating, which may trade-off the treatment effect. In the proposed hypnosis method, self-control is not needed for patients during the whole treatment procedure. Moreover, under the guidance of hypnotist, the patient could stay in peace state and feel comfortable during treatment.

**Figure 7:**
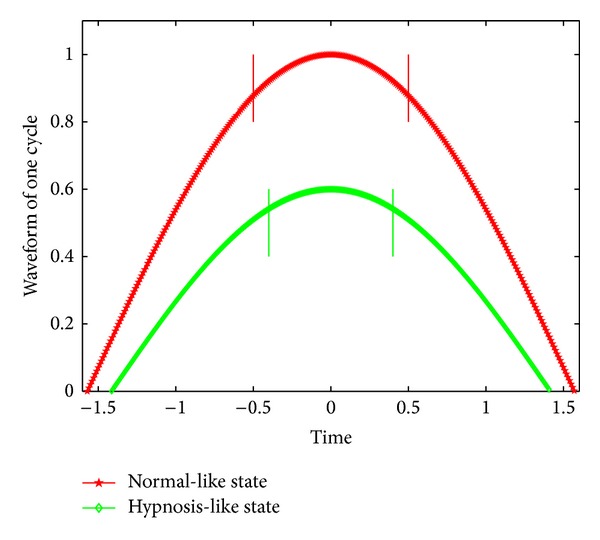
Comparison of waveforms between the hypnosis-like state (green) and the normal-like state (red); the green curve has the more stable peak than the red curve, caused by the amplitudes and the cycles of two curves.

It is clear that the amplitude nearby wave peak in the hypnosis state is more stable than that in the normal state, because the mean peak in the hypnosis state is much lower than that in the normal state as shown in Figure 2. If the waveforms of the two states are similar, the mean cycles for two states should have the same proportion with the mean peaks. However, the difference of mean cycle between two states is not notable as shown in Figure 3. This leads to more slow reduction of amplitude under the hypnosis state than that under the normal state, as shown in Figure 7. Again, it demonstrates the stability of respiration under the hypnosis state.

Suppose the tumor in the lung rigidly moves with respiration motion, when the tumor is irradiated at the peak of a respiration cycle, the dose in tumor is determined by the mean ratio between the displacement with time weight and the amplitude, which is the definition of ratio in (2). If the value of ratio is “1” during the whole cycle, apparently the waveform is square wave, which is an ideal state without tumor motion. 

In the experiment, six volunteers were selected. It may arouse controversy that the quantity is not enough, and the age or other properties are not generally representative. However, differences always exist between the volunteers and the patients. The major contribution of the proposed method is the use of hypnosis during radiotherapy. One of the challenging problems is how to protect the hypnotist from radiation exposure, which is a key technology for the method. The ultimate goals of the method are to treat the patients with least side effects and to become applicable to a larger number of patients.

Gamma ratio is a parameter to evaluate the similarity of two distributions. As it is widely used in evaluation of dose distributions, we extend the use of gamma ratio to evaluate the difference between two respiration waveforms which may cause the change of dose distribution during radiotherapy. Therefore, we choose the gamma ratio as the evaluation metrics, although other parameters can also describe the difference.

This work is a preliminary clinical study instead of a clinical study. However, it is necessary before the further clinical application. Although it is limited to the establishment of the relationship between hypnosis and respiratory motion, this establishment has demonstrated the feasibility of the clinical application of hypnosis during radiotherapy.

## 5. Conclusion

In this work we found that the hypnosis can effectively stabilize respiration motion, which makes it suitable to control respiration during radiotherapy of lung cancer. Although hypnosis is not applicable to all cases, it provides an alternative way for respiratory control without any side effects. The next problem is how to use hypnosis in clinical conditions. We will propose a new scheme of respiratory control using hypnosis in radiotherapy of lung cancer. Since it is a noninvasive method, hypnosis intervention will find various clinical applications in the near future.

## Figures and Tables

**Table 1:** Physiological parameters of the six volunteers.

Parameters*∖*volunteer no.	1	2	3	4	5	6

Age (year)	22	22	23	27	23	28

Weight (kg)	56	50	60	55	52	73

Chest circumference (cm)	89	85	91	88	75	100
